# Attrition and social vulnerability during 2-year-long structured care in type 2 diabetes, the ERMIES randomized controlled trial

**DOI:** 10.1186/s12902-022-01211-3

**Published:** 2022-12-13

**Authors:** Anna Flaus-Furmaniuk, Adrian Fianu, Victorine Lenclume, Emmanuel Chirpaz, Maryvette Balcou-Debussche, Xavier Debussche, Catherine Marimoutou

**Affiliations:** 1 Endocrinology Department, University Hospital of the Reunion Island, Saint Denis, France; 2 INSERM CIC 1410, University Hospital of the Reunion Island, Saint Denis, France; 3 CERPOP, Université de Toulouse, Inserm, UPS, Toulouse, France; 4 Icare Research Unit, Institut Coopératif Austral Pour La Recherche en Éducation EA7389, University of Reunion, Saint-Denis, La Réunion, France

**Keywords:** Type 2 diabetes, Self-management education, Health literacy, Clinical trial, Attrition

## Abstract

**Background:**

Diabetes self-management education is exposed to attrition from services and structured ambulatory care. However, knowledge about factors related to attrition in educational programs remains limited. The context of social vulnerability due to low income may interfere. The aim of this study was to identify the sociodemographic, clinical, psychometric, and lifestyle factors associated with attrition from the ERMIES multicentre randomized parallel controlled trial (RCT) that was interrupted due to the combination of both slow inclusion and high attrition.

**Methods:**

The ERMIES trial was performed from 2011 to 2016 on Reunion Island, which is characterized by a multicultural population and high social vulnerability. The original objective of the RCT was to test the efficacy of a2-year structured group self-management education in improving blood glucose in adult patients with nonrecent, insufficiently controlled type 2 diabetes. One hundred participants were randomized to intensive educational intervention maintained over two years (*n* = 51) versus only initial education (*n* = 49). Randomization was stratified on two factors: centres (five strata) and antidiabetic treatment (two strata: insulin-treated or not). Sociodemographic, clinical, health-care access and pathway, psychometric and lifestyle characteristics data were collected at baseline and used to assess determinants of attrition in a particular social context and vulnerability. Attrition and retention rates were measured at each visit during the study. Multiple correspondence analysis and Cox regression were performed to identify variables associated with attrition.

**Results:**

The global attrition rate was 26% during the study, with no significant difference between the two arms of randomization (9 dropouts out of 51 patients in the intervention group and 17 out of 49 in the control group). Male gender, multiperson household, low household incomes (< 800 euros), probable depression and history of hospitalization or medical leave at inclusion were associated with a higher risk of attrition from the study in multivariate regression.

**Conclusions:**

Social context, vulnerability, and health care history were related to attrition in this 2-year longitudinal comparative study of structured care. Considering these potential determinants and biases is of importance in scaling up interventions aimed at the optimization of long-term care in type 2 diabetes mellitus.

**Trial registration:**

ID_RCB number: 2011-A00046-35, Clinicaltrials.gov number: NCT01425866 (Registration date: 30/08/2011).

Source of funding: Ministry of Health, France.

## Background

Type 2 diabetes mellitus (T2DM) is a major health problem that results from a complex interaction between polygenic inheritance and social, lifestyle, and environmental factors and affects both developed and developing countries. In 2019, 463 million people and 9.3% of 20- to 79-year-old adults were estimated to be diabetic worldwide [1]. T2DM was identified by the Global Burden of Disease Study 2017 in the top ten major causes of reduced life expectancy and resulted in 1.37 million deaths worldwide in 2017 (+ 43% versus 2007) [2]. In 2015, the prevalence of pharmacologically treated diabetes in France was estimated at 5.0% from the French national health insurance information system, with large regional disparities. French overseas departments had a higher prevalence, the highest being Reunion Island, with 10.2% of people treated for diabetes [3].

Reunion Island is located in the southwestern Indian Ocean. It has a highly multicultural population with a crossbreeding of different ethnic groups (Creole, Malagasy, African, Indian, European, and Chinese) and high social vulnerabilities compared to metropolitan France: a higher proportion of single-parent families (18% vs. 8%), early motherhood < 20 years (23% vs.4%), and unemployment rate (29% vs. 10,2%). Approximately 42% of the population lives below the French poverty line of €935/month [4]. Disparities in access to adequate food, exercise, health care and services have been underlined [5]. This may explain why, despite identical standards of care, the glycaemic control in T2DM patients in Reunion Island remained suboptimal, with a mean haemoglobin A1c (HbA1c) of 7.4% versus 7.1% in continental France according to an ENTRED declarative study conducted in 2007 [6]. Reunion Island has experienced some deep evolution in the economic, social, cultural, and health domains since 1970. These changes were inspired by the willingness to catch up with the standards of continental France [7]. Despite many positive transformations, the Reunionese population remains vulnerable and struggles with a high proportion of health and social disparities. Reunion Island also experiences high attrition from medical care in diabetes-treated patients. Recently, the Regional Health Observatory of Reunion Island published data from the longitudinal analysis (2010–2018) of the health care pathway of patients with pharmacologically treated diabetes issued from the regional health insurance information system [8]. Among 3 597 patients who started medical treatment of diabetes in 2010, the quarterly follow-up by general practitioners, recommended by the national guidelines, was missing in 19% of patients after two years and in 40% of patients after eight years of follow-up. Similarly, 36% of the patient cohort interrupted their medications at least for a while during the study period [8].

Large intervention trials in T2DM have shown that complications can be prevented or delayed by rigorous control of blood sugar levels and risk factors [9, 10]. Recent guidelines highlighted the need for a patient-centred personalized approach [11] based on individualized glycaemic targets. To achieve these goals, patients should adhere to and continuously adjust their pharmacological treatment as well as adapt their lifestyles and nutritional habits [12]. This global approach requires patients’ active participation in disease management and coping with multiple challenges related to self-management. Diabetes self-management education and support (DSME) provides help for patients to acquire or maintain the skills they need for decision-making. DSME links the necessary knowledge for disease management, patient environment, and health literacy [13, 14]. Health literacy refers to the cognitive and social skills that determine the motivation and ability of individuals to gain access to, understand, retrieve, and use information in ways that promote and maintain good health [15]. Health literacy has been linked to numerous health indicators and outcomes [16] and is a key component of health perceptions and practices [17–19].

It has been shown in previous studies that DSME can improve HbA1c by as much as 1% and has a positive effect on psychological, social, and behavioural aspects of diabetes [20–23]. However, whereas the effects of structured education have been largely demonstrated in short- and mid-term settings, data are less conclusive after one year of follow-up. The main concern about long-term effects is the risk of attrition during the program. A recent large systematic review clearly showed that a significant change in Hba1c following DSME was associated with higher subject retention rates [24]. However, our knowledge about factors related to attrition in educational programs remains limited, and the context of health and social transition, as observed on Reunion Island, may interfere. Randomized controlled trials (RCTs), especially those focusing on DSME, can provide solid data on patients’ sociodemographic or clinical characteristics and lifestyle associated with attrition, which may help understand or prevent attrition during follow-up in studies such as clinical practice. The study of attrition and potential related factors in clinical trials targeting the effectiveness of DSME interventions can help identify individual profiles requiring additional support to prevent attrition from DSME programs. It could also contribute to the understanding of the underlying mechanism of attrition during interventions in vulnerable populations and in contexts of socioeconomic transition.

The aim of the present work was to identify the sociodemographic, clinical, psychometric, and lifestyle factors associated with attrition in a two-year RCT comparing two different schedules of educational interventions in Reunion Island between 2011 and 2016 (ERMIES RCT).

## Methods

### Sample and setting

ERMIES was a multicentre randomized two-arm controlled trial that tested the efficacy of a long-term (two years) structured group self-management educational intervention in improving blood glucose in nonrecent, insufficiently controlled T2DM patients compared to a 3-month initial education course only. The detailed protocol of the trial (NCT01425866 ClinicalTrials.gov) is described elsewhere [25].

The main eligibility criteria included age ≥ 18 years, type 2 diabetes treated for more than one year, HbA1c ≥ 7.5% for ≥ 3 months, without any severe currently evolving complication (ischaemic or proliferative retinopathy, severe chronic renal insufficiency (clearance < 15 ml/min), coronary heart disease, foot lesions), and absence of any major physical or cognitive handicap. After written consent was obtained, participants were randomized to either the intervention or control arm of the study (allocation ratio 1:1). Computing randomization was stratified on 2 factors: centres (five strata) and antidiabetic treatment (two strata: insulin-treated or not).

The calculated necessary number of subjects to include in the study was 99 per arm. It was increased to 120 for taking into account an estimated data deficiency of 20% at two years (drop out, refusals, deaths), making a total number to include 240 subjects [25].

The trial design included an initial group education course conducted by trained educators blinded to the subsequent group allocation within the 12 weeks following inclusion. At the end of these 12 weeks, patients allocated to the intervention group were invited to receive ongoing structured education within group sessions (3–10 patients) for 90 to 120 min each at 16, 32, 48, 64, 80, and 96 weeks. In the control arm, patients did not follow any further structured education but received a quarterly medical consultation in a diabetes specialized medical unit up to the 96th week.

Patients were reminded by telephone during the week prior to each educational session or medical consultation to confirm the date, time, and place. Patients who failed to attend the session or medical visit were called within two days and offered a new schedule.

Finally, between 2011 and 2016, only 100 patients could be included in the ERMIES trial due to difficulties in patient enrolment, denying the possibility of analysing the main outcome (1% decrease in HbA1c at two years). These 100 patients constituted the present study baseline sample.

### Measures

Attrition and retention rates were measured at each visit during the study. Whenever possible, reasons for withdrawing from the study were gathered directly. The adverse events motivating attrition were analysed individually from the original data and completed through self-administered questionnaires.

Clinical features and anthropometric indicators (body mass index, waist circumference) were assessed by physicians or nurses. Data collected at baseline were grouped into five categories: sociodemographic, clinical, health care access and pathway, lifestyle, and psychometric scales. Adherence to treatment was evaluated using the Compliance Evaluation Test (CET) as described by Girerd et al. [26]. Health practices (level of physical activity and food consumption) were assessed by questionnaires used in Reunion Island for a number of descriptive or intervention studies: RECONSAL [27], REDIA-prev1 [27], and REDIA-prev2 [28]. Regular physical activity was assessed using a questionnaire derived from Baecke et al. [29]. Professional and home physical activity scores were based on the sum of five items dealing with the frequency of sitting, standing, walking, and lifting heavy loads (five-point scale score). The notion of sweating during activity was not taken into account due to the tropical geographic localization of Reunion Island. Sedentary lifestyle was defined by a score < 13/20 (median value). Food consumption (reported energy intake, macronutrient intake, dietary habits) was assessed by means of a rapid food frequency questionnaire relating to weekly consumption [30]. The questionnaire was conducted face to face with the centre nurse trained for this purpose before the start of the research. The quantities of various foods are assessed by means of a photo album. The validity of the questionnaire and the photo album for the population of Reunion was checked by comparison with food surveys. Food balance was evaluated via a score (0–6) based on the sum of the answers to three questions (scored 0–2 points): “how often do you eat fry food?” (“never” = 0, “ < 4x/week” = 1 and “ >  = 4x/week" = 2), “how often do you have some extralarge meals (party, restaurant, family meeting, etc.)?” (“never” = 0, “ <  = 2/month” = 1, “ > 2/month” = 2), and “do you eat snacks between meals?” (“never” = 0, “sometimes” = 1, “frequently” = 2). An unbalanced diet was defined by a score > 3. Cut-offs for waist circumference were > 88 cm (female) or > 102 cm (male).

Self-efficacy, social support, and anxious depressive state were assessed by means of psychometric scales validated for the purpose of the trial in Reunion [25]. Four psychometric scales (including six subscales) were used:Quality of life was assessed by two subscales suitable for T2DM: satisfaction with diabetes control (six items; range 0–4) and reported adherence with self-care regimen (six items; range 0–4) from the DQOL-BCI (Diabetes Quality of Life, Brief Clinical Inventory) [31]. The Multidimensional Diabetes Questionnaire (MDQ) was used to assess self-efficacy (seven items; range 0–4), outcome expectancies (six items; range 0–4), positive reinforcing behaviours (eight items; range 0–3), and misguided support behaviours (four items; range 0–3) [32]. The results for MDQ and DQOL-BCI questionnaires were classified as “high” if superior to the median value for the item. Patient anxiety was assessed by seven items from the HADS (Hospital Anxiety and Depression Scale), with a cut-off for anxiety risk of > 11 according to the literature [33]. The depression level was measured by means of the CES-D centre for epidemiologic studies depression scale (range 0–60) with the cut-off for probable depression fixed at > 16 according to the literature [34, 35]. HbA1c was assessed both at baseline and at 96 weeks (measured by the HPLC method in a centralized biochemistry laboratory of the Félix Guyon Hospital, St Denis, Reunion).

### Analyses

The completeness and accuracy of the data were double checked during data recording on the Ennov Clinical software from the paper questionnaire and then by consistency tests programmed by the data manager (Ennov Clinical V.7.5, Ennov Group, Paris, France). The initial dataset for regression analysis comprised 27 baseline variables (i.e., measured at the trial’s entry) including 19 variables with at least one missing observation but never more than 11.

To minimize selection bias due to incomplete data at baseline, we used a multiple imputation strategy under missing-at-random assumption. As the multivariate description of the variables highlighted an arbitrary missing-value pattern (data not shown), we selected the multivariate imputation using chained equations to impute baseline missing data using the mi impute command from Stata software (version 13.1). We performed 42 imputations, as 42 patients presented at least one missing value.

The main criterion of this study was attrition. Patients were classified into the attrition group if they quit the study between inclusion and week 96 and compared to those who completed the whole follow-up using the chi-square test for categorical variables, the independent sample t test for continuous variables, or the Mann–Whitney U test for nonnormally continuous variables. The Kaplan‒Meier survival curve of retention was stratified according to randomization status and compared using the log-rank test.

To identify baseline characteristics and profiles associated with attrition, an exploratory Multiple correspondence analysis (MCA) analysis to select variables to implement in the regression model. MCA was performed separately in five categories: sociodemographic, clinical, health care access and pathway, lifestyle, and psychometric scales.

From the 112 variables from the ERMIES database, 17 variables were combined into 4 scores regarding health practices (dietary balance, physical activity separately at work and at home, checked as described above, and smoking (Y/N)), 64 variables were combined into the 4 psychometric scales (including 6 subscales) described in the previous paragraph, and 43 variables of adjustment were included in the MCA. The MCA performed separately for sociodemographic, clinical, health care access and pathway, lifestyle characteristics and psychometric scales allowed us to eliminate 16 variables potentially duplicating or not relevant, leaving 27 candidate variables to include in the regression models after the multiple imputation for missing data.

The selection of the initial multivariate model was based on a bivariate analysis statistical significance level of 20%. Backwards elimination was then used to select variables for the final model with a *p* value of 5%. The hazard ratio (HR) in the Cox regression model estimated the relative likelihood of attrition during the study. The proportional hazards assumption was checked by Schoenfeld tests of residuals.

The statistical analyses for descriptive analysis, MCA, Kaplan‒Meier were performed using SAS version 9.4 software (SAS Inc., Cary, NC, USA). Cox proportional hazards regression analysis and linear regression analysis were performed using STATA version 13.1 software (StataCorp. 2013. Stata Statistical Software: Release 13. College Station, TX: StataCorp LP).

### Ethical consideration

The ERMIES RCT was registered in the European RCT database in August 2011. It received approval from the ethics committee, “Comité de Protection des Personnes (CPP) Sud-ouest et Outre Mer III”, and authorization from the French Health Products Safety Agency (Agence française de sécurité sanitaire des produits de santé, Afssaps). Data were computerized according to the reference methodology for clinical trials (MR 001) of the French commission on freedom of information (CNIL). The present study used the computerized anonymous data of the patients who signed an informed consent to participate in the ERMIES study.

## Results

**Fig. 1:**
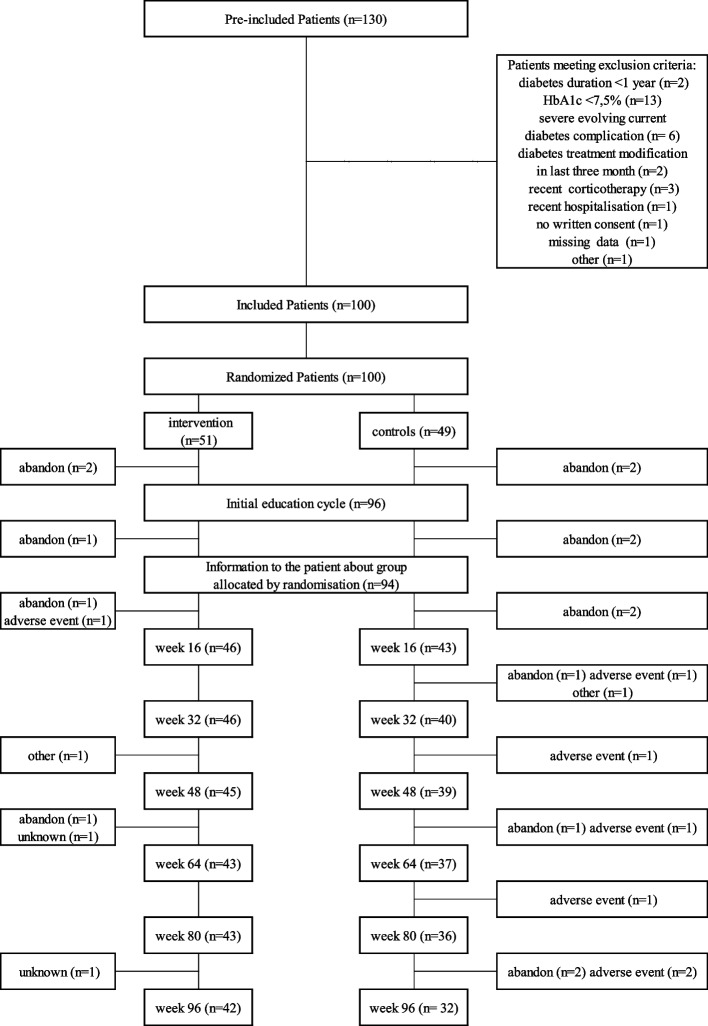
Attrition diagram according to ERMIES trial design involving two parallel groups

Patients were recruited from the four public hospitals of Reunion Island from October 2011 to November 2014 and followed up to December 2016. Of the 100 participants included in the trial, 26% dropped out, and 74% completed the study. The attrition of patients according to intervention design is presented in Fig. 1. In the intervention group, the attrition was 9 out of 51 patients. It was 17 out of 49 in the control group, including 6 withdrawals for adverse events (four of them were hospitalizations for uncontrolled diabetes) versus one in the intensive education group. However, the survival curve of the attrition did not significantly differ between the two groups. The Kaplan‒Meier survival curve of retention probability was not significantly different between the two education groups.

Tables 1 and 2 show thepatients’ characteristics at baseline and according to attrition. Two-thirds of the participants were female; half of the population had a low educational level (primary school). A third of the population was active in,line with amedian age of 59 years [interquartile range (IQR) 52.5–65 years]. Almost 90% of patients were overweight or obese. The median HbA1c was 8.8% [IQR 8.2–9.6]. There was no significant difference between patients in the attrition group and patients who completed the study with regard to demographic, clinical, lifestyle and health-care access characteristics except for age (the attrition group was significantly younger) and the use of nonemergency medical transportation (more frequently used in the attrition group). There were some differences in conditions of living according to age: 70% of patients aged < 60 lived in households with two or more people, whereas 38.8% of those aged ≥ 60 lived alone, 42.8% lived with one person, and 18.4% lived with at least two people. The quality of life assessed with the DQOL-BCI Diabetes Quality of Life showed a median score of 2.7 [IQR 2.2–3.2] for satisfaction with diabetes control and 2.3 [IQR 2.0–3.0] for reported adherence to the self-care regimen. According to theCES-D and HADS, 50% of participants presented with possible depression and anxiety. Social support analysis showed a median of positive reinforcing behaviours of 1.9 [IQR 0.6–2.7] points (range 0–3) and 0.6 [IQR 0–1.6] points (range 0–3) for misguided support behaviours. There was no significant difference according to attrition. Among the 72 patients with a complete follow-up, available baseline and final HbA1c values (*n* = 72), the median individual change in HbA1c was -0.9% [IQR -1.5 to -0.25].

**Table 1:** Sociodemographic, clinical, lifestyle, and health-care access patients’ baseline characteristics (*N* = 100)

	Total		Complete follow-up		Attrition group		*p* value*
	% miss	n (%)	% miss	n (%)	% miss	n (%)	
Age, years; median [IQR]	-	59 [52.5–65]	-	60 [55–66]	-	56 [45–65]	**0.002**
Female	-	67 (67.0)	-	53 (71.6)	-	14 (53.9)	0.097
T2DM duration, years; median [IQR]	-	15 [10–24]	-	16 [11–24]		13 [8–20]	0.528
Chronic complications **	-	57 (57.0)	-	41 (55.4)	-	16 (65.4)	0.587
Microvascular		45 (45.0)		33 (44.6)		12 (46.1)	0.891
Macrovascular		33 (33.0)		21 (28.4)		12 (46.1)	0.097
Education level	-		-		-		0.242
≤ Primary school		48 (48.0)		39 (52.74)		9(34.6)	
Middle school		31 (31.0)		20 (27.0)		11 (42.3)	
≥ Secondary school		21 (21.0)		15 (20.3)		6 (23.1)	
Cohabitation status	1		1.3		-		0.083
Living alone		26 (26.3)		23 (31.5)		3 (11.5)	
Living with 1person		29 (29.3)		18 (24.7)		11 (42.3)	
Living with 2 or more		44 (44.4)		32 (43.8)		12 (46.2)	
Employment status	1		1.3		-		0.553
Part/full-time		27 (27.3)		23 (31.5)		11 (42.3)	
Retired		39 (39.4)		20 (27.4)		7 (26.9)	
Unemployed		34 (34.3)		30 (41.1)		8 (30.8)	
Gross monthly household income	-		-		-		0.063
< 800 euros		33 (33.0)		21 (28.4)		12 (46.2)	
800–1999 euros		43 (43.0)		37 (50.0)		6 (23.1)	
> 1999 euros		22 (22.0)		15 (20.3)		7 (26.9)	
unknown		2 (22.0)		1 (1.4)		1 (3.8)	
Universal Health Coverage "CMU"	-	39 (39.0)	-	28 (37.8)	-	11 (42.3)	0.687
BMI class	-		-		-		0.972
18.5–24.9		13 (13.0)		10 (13.5)		3 (11.5)	
25–29.9		43 (43.0)		31 (41.9)		12 (46.2)	
> = 30		44 (44.0)		33 (44.6)		11 (42.3)	
BMI. kg/m^2^ [median [IQR)]	-	28.6 [26.5–33.6]	-	28.6 [26.6–34.8]	-	28.9 [26.4–32.2]	0.224
Increased waist circumference ^a^	1	74 (74.8)	1.3	56 (76.5)	-	18 (69.2)	0.451
HbA1c. % median [IQR]	-	8.8 [8.2–9.6]	-	8.7 [8.1–9.6]	-	9.0 [8.4–9.6]	0.899
Antidiabetic treatment	4		1.3		12.5		0.947
insulin ± oral treatment		59 (61.5)		45 (61.6)		14 (60.9)	
oral treatment		37 (38.5)		28 (38.4)		9 (39.1)	
Dietary imbalance ^b^	1	56 (56.6)	1.3	44 (60.3)	-	12 (46.1)	0.212
Physical activity at home ^c^	7		6.7		8.3		0.405
sedentary		57 (61.3)		44 (63.8)		13 (54.2)	
Physical activity at work ^d^	5		6.7		-		0.341
Unemployed		67 (70.5)		49 (71.0)		18 (69.2)	
Sedentary work		15 (15.8)		9 (13.0)		6 (23.1)	
Active work		13 (13.7)		11 (15.9)		2 (7.7)	
Smoking	-		-				0.530
Nonsmoker		63 (63.0)		49 (66.2)		14 (53.9)	
Smoker		9 (9.0)		6 (8.1)		3 (11.5)	
Former smoker		28 (28.0)		19 (25.7)		9 (34.6)	
NEMT in past year	5	24 (25.3)	5.3	14 (20.0)	4.2	10 (40.0)	**0.048**
Medical leave or hospitalization in past year	2	32 (32.7)	1.3	20 (27.4)	4.2	12 (48.0)	0.058
Treatment compliance ^e^	9		6.7		16.7		0.950
Good		18 (19.8)		14 (20.3)		4 (18.2)	
Partially		47 (51.6)		35 (50.7)		12 (54.5)	
Noncompliant		26 (28.6)		20 (30.0)		6 (27.3)	
GP and diabetologist follow-up	5		5.3		4.2		0.876
< 3 visits/year		16 (16.8)		12 (17.1)		4 (16.0)	
3–4 visits/year		56 (58.9)		42 (60.0)		14 (56.0)	
> 4 visits/year		23 (24.2)		16 (22.9)		7 (28.0)	

Data are relative frequencies – n (%) for categorical variables and medians and interquartile range [IQR] for continuous variables

Abbreviations: *N* Sample size, *% miss* Percentage of missing values, *IQR* Interquartile range, *NEMT* Nonemergency medical transportation, *GP* General practitioner, *BMI* Body mass index

*Chi-square test for categorical and independent sample t test for continuous variables, respectively ^**^ chronic complications correspond to history of stable chronic condition at baseline while presenting a severe evolving current complication at baseline was a criteria of noninclusion in ERMIES RCT

^a^Waist circumference > 88 cm (female) or > 102 cm (male)

^b^dietary score (0–6), dietary imbalance if > 3

^c^Physical activity at home score (5–20), sedentary if < 13/20

^d^Physical activity at work score (5–20), sedentary if < 13/20

^e^Treatment compliance score (0–6), 0 = good, 1–2 partially, >  = 3 noncompliant

**Table 2:** Psychometric scales at baseline (*N* = 100)

	Total		Complete follow-up		Attrition group		*p* value
	% miss	median [IQR]	% miss	median [IQR]	% miss	median [IQR]	
**DQOL-BCI**							
Satisfaction with diabetes control (6 items; range 0–4)	11	2.7 [2.2–3.2]	12.1	2.7 [2.2–3.0]	7.6	2.8 [2.3–3.2]	0.448°
Reported adherence with self-care regimen (6 items; range 0–4)	12	2.3 [2.0–3.0]	13.5	2.3 [2.0–2.9]	7.6	2.7 [2.0–3.0]	0.410*
**MDQ**							
Self-efficacy (7 items; range 0–4)	9	2.7 [2.1–3.3]	9.4	2.7 [2.1–3.4]	7.6	2.7 [2.3–3.1]	0.797*
Outcome expectancies (6 items; range 0–4)	6	4.0 [3.7–4.0]	5.4	4.0 [3.7–4]	7.6	4.0 [3.8–4.0]	0.193*
Positive reinforcing behaviours (8 items; range 0–3)	12	1.9 [0.6–2.7]	14.8	1.9 [0.5–2.6]	3.8	2.0 [0.8–2.8]	0.591*
Misguided support behaviours (4 items; range 0–3)	4	0.6 [0–1.6]	5.4	0.5 [0.0–1.3]	-	1.0 [0.0–2.0]	0.210*
**HADS: Hospital Anxiety and Depression scale (range 0–21)**	3	11.0 [9.0–13.0]	2.7	11.0 [9.0–13.0]	3.8	12.0 [9.0–12.0]	0.271°
**CES-D: centre for epidemiologic studies depression scale (range 0–60)**	8	16.0 [10.0–26.5]	9.4	15.0 [10.0–27.0]	3.8	21.0 [15.0–26.0]	0.284*

Data are medians and interquartile range [IQR]

Abbreviations: *N*—Sample size, *IQR* Interquartile range, *% miss* Percentage of missing values, *DQOL-BCI* Diabetes quality of life brief clinical inventory, *MDQ* Multidimensional diabetes questionnaire

* Mann–Whitney U test or °independent sample t test

The Cox regression analyses of factors associated with attrition are presented in Table 3. In the univariate analysis, depression was the only variable that reached significance (HR 2.52. However, male gender (vs. female), two-person household (vs. living alone), low income (vs. intermediate income), and medical leave or hospitalization in within the past year were found to be significantly associated with attrition in the final multivariate model. Depression remained significantly associated after adjustment for HR = 4.04. There was no clinical or lifestyle variable significantly associated with attrition.

**Table 3:** Factors associated with attrition in the ERMIES study (Cox regression analysis)

			Univariate					Multivariate			
Factor	Category/Class		HR		95% Cl		*P* value	HR	95% Cl	*P* value	
**Sociodemographic characteristics**											
**Age**							0.23				
	18-60 years		Reference								
	≥ 60 years		0.61		[0.28 to 1.35]						
**Sex**							0.13			**0.02**	
	male		Reference					Reference			
	female		0.55		[0.25 to 1.19]			0.30	[0.11 to 0.81]		
**Universal health coverage "CMU"**							0.69				
	no		Reference								
	yes		1.17		[0.54 to 2.55]						
**Education**							0.24				
	Primary school (or less)		Reference								
	Middle school		2.13		[0.88 to 5.15]						
	Secondary school (or more)		1.53		[0.54 to 4.31]						
**Cohabitation status**							0.10			**0.02**	
	Living alone		Reference					Reference			
	Living with 1person		3.91		[1.09 to 14.05]			7.09	[1.84 to 27.37]		
	Living with 2 or more		2.43		[0.69 to 8.63]			3.32	[0.88 to 12.56]		
**Gross monthly household income**							0.08			**0.01**	
	<800 euros		Reference					Reference			
	800-1999 euros		0.34		[0.13 to 0.90]			0.17	[0.06 to 0.54]		
	>1999 euros		0.85		[0.34 to 2.16]			0.62	[0.22 to 1.72]		
	unknown										
**Clinical features**											
**BMI**							0.70				
	<30		Reference								
	≥30		0.86		[0.39 to 1.87]						
**Antidiabetic treatment**							0.89				
	oral treatment		Reference								
	insulin +/- oral treatment		0.94		[0.42 to 2.14]						
**Increased waist circumference**^a^							0.50				
	No		Reference								
	Yes		0.75		[0.33 to 1.72]						
**Hba1c class**							0.54				
	≤8.2		Reference								
	8.2-8.8		1.75		[0.56 to 5.53]						
	8.8-9.6		2.30		[0.75 to 7.04]						
	>9.6		1.60		[0.49 to 5.24]						
**Lifestyle **											
**Dietary imbalance**^b^							0.15				
	No		Reference					-	-	**-**	
	Yes		0.57		[0.26 to 1.23]						
**Physical activity at home**^c^							0.34				
	Sedentary		Reference								
	Active		1.48		[0.67 to 3.27]						
**Physical activity at work**^d^							0.37				
	Unemployed		Reference								
	sedentary work		1.58		[0.62 to 4.02]						
	active work		0.53		[0.12 to 2.29]						
**Smoking**							0.54				
	Non-smoker		Reference								
	Smoker		1.67		[0.48 to 5.83]						
	former smoker		1.50		[0.65 to 3.47]						
**Health care access and pathway**											
**NEMT in past year**							0.08				
		No						-			
		Yes		2.07	[0.93 to 4.6]						
**Medical leave or hospitalization in past year**							0.05				**0.03**
		No		Reference				Reference			
		Yes		2.19	[1.00 to 4.79]			2.75	[1.13 to 6.72]		
**Treatment compliance**^e^							0.98				
		Good		Reference							
		Partially		1.11	[0.36 to 3.36]						
		noncompliant		1.05	[0.30 to 3.66]						
**GP and diabetologist follow-up**							0.82				
		<3consults/year		Reference							
		3-4 consults/year		1.05	[0.34 to 3.23]						
		> 4consults/year		1.38	[0.40 to 4.80]						
**Psychometric scales**											
**DQOL-BCI**											
**Satisfaction with diabetes control (6 items)**							0.27				
		low ^f^		Reference							
		High ^g^		1.56		[0.71 to 3.44]					
**Reported adherence with self-care regimen (6 items)**							0.11				
		low ^f^		Reference				-	-		**-**
		high ^g^		1.99		[0.85 to 4.69]					
**CES-D: centre for epidemiologic studies depression scale**							0.04				**0.01**
		normal score		Reference				Reference			
		at risk for clinical depression		2.52		[1.05 to 6.06]		4.04	[1.35 to 12.02]		
**HADS: Hospital Anxiety and Depression scale**							0.80				
		normal score		Reference							
		probable mood disorder		0.90		[0.41 to 1.98]					
**MDQ Multidimensional diabetes questionnaire**											
Outcome expectancies and social support in the self-care behaviours							0.18				
		low ^f^		Reference				-	-		**-**
		high ^g^		1.83		[0.76 to 4.38					
Self-efficacy (7 items)							0.70				
		low ^f^		Reference							
		high ^g^		1.17		[0.53 to 2.57]					
Misguided support behaviours (4 items)							0.18				
		low ^f^		Reference				-			
		high ^g^		1.74		[0.78 to 3.85]					
Positive reinforcing behaviours (8 items)							0.67				
		low ^f^		Reference							
		high ^g^		1.19		(0.54 to 2.62]					
**Intervention status**											
**Randomization group**							0.07				
		regular visits		Reference				-			
		intensive educational program		0.48		[0.21 to 1.07]					

Data are hazard ratios (HRs) with 95% confidence intervals (95% CIs) and *P* values for the global effect of the factor. All results were obtained after multiple imputation for missing baseline data.

Abbreviations: *HR* Hazard ratios, *95% CI—95%* Confidence interval, *NEMT* Nonemergency medical transportation, *GP* General practitioner, *BMI* Body mass index, *IQR* Interquartile range, *DQOL-BCI* Diabetes quality of life brief clinical inventor

^a^Waist circumference > 88 cm (female) or > 102 cm (male)

^b^dietary score (0–6), dietary imbalance if > 3

^c^Physical activity at home score (5–20), sedentary if < 13/20

^d^Physical activity at work score (5–20), sedentary if < 13/20

^e^Treatment compliance score (0–6), 0 = good, 1–2 partially, >  = 3 noncompliant

^f^< 50^th^ percentile

^g^> 50^th^ percentile

## Discussion

The aim of this study was to identify the sociodemographic, clinical, psychometric, and lifestyle factors associated with attrition from the ERMIES multicentre RCT. In this DSME two-year follow-up trial, a global attrition rate of 26% was observed, with no significant difference between the two arms. Factors found to be significantly associated with attrition in the final multivariate model were male sex, depression, low household incomes (< 800 euros), and history of hospitalization or medical leave in the year before patient inclusion in the RCT.

To our knowledge, this is the first study in France, as well as in countries affected by socioeconomic transition, focused on the attrition factors in intervention trials focused on self-management education. This is of importance to the necessity of ongoing self-management support over years through the diabetes medical story to achieve long-term positive outcomes. On Reunion Island, the first DSME trial in T2DM, REDIAprev2, was conducted in 2004–2005. It aimed to compare quarterly individual lifestyle counselling visits by a registered nurse and a dietitian (intervention group) with usual care (control group). The global attrition was 20% at the12-month follow-up, which was higher in the intervention group (26%) than in the control group (14%, *p =* 0.002,) with 77% of attrition occurring before the second visit. Notably, patients in the control group had more medical appointments: 30.3% vs. 18.7% in the intervention group (*p* < 0.05). However, the factors associated with attrition were not investigated [28].

According to a recent systematic review, the retention rates were >  = 80% in 84 out of 118 (71%) DSME interventions and < 80% for 22 (19%); 12 interventions presented insufficient data to determine retention [24]. In regard to these results, our retention rate of 74% is consistent. Our study population was characterized by a fairly high level of social vulnerability, as evidenced by the proportion of participants who were unemployed or retired, had low income, lived alone, or had a low level of education. Most of the participants had been diabetic for more than 10 years and presented ahad chronic complications. Studies assessing self-management education have seldom specified baseline indicators of vulnerability. Most studies included either newly diagnosed T2DM patients or participants with a T2DM duration less than 10 years [36]. The most frequently reported socioeconomic characteristics were education level and employment status [37–39]. The UK Diabetes Manual trial enrolled patients from practices in anurban multiethnic and socioeconomically deprived population, with a retention rate of 72% in the intervention group and 85% in the control group at six months [40].

In the present study, attrition was higher in males. In previous studies on diabetes in Reunion Island, a tendency to lower the inclusion rate of men versus women had been noticed [27, 28, 41], leading to an overrepresentation of women in regard to the proportion of females in the national health insurance data, the prevalence of declared and/or treated diabetes on Reunion Island was higher in women than in men (9.6% versus 7,9%) [42]. On the other hand, a study performed in a random sample of 3600 subjects aged 30–69 years showed that when considering undetected cases, diabetes was slightly more frequent in males (17.7 vs. 17.1%) [41]. Taken together, these data suggest a possible underdiagnosis and lower seeking of care in men with T2DM on Reunion Island. Moreover, similar data were observed during the REDIA-prev1 (REunion DIAbetes primary prevention) cohort study implemented in 2010–2011. Nine years after inclusion, a high rate of attrition was observed (42%), with an overrepresentation of men and younger participants among the attrition group [43]. This could be due to easier or more effective access to the health-care system in T2DM women than in T2DM men. We have not found any literature on health care seeking in French diabetic women, but the Prevalence of Hypertension among Disadvantaged Guadeloupeans study performed in another French overseas department between 2003 and 2014 did observe an increase in both hypertension awareness and the proportion of treated individuals in women compared to men [44]. Moreover, according to the analysis of patients’ access to health care and medicines across low-income countries performed by Srivastava et al., female sex was one of the main determinants of health-seeking behaviour [45].

Patients who completed the study were significantly older at baseline (*p =* 0.002); however, they had less frequently used nonemergency medical transport (NEMT) in the year before the study than in the attrition grouip (*p =* 0.048). Moreover, they were almost twice as often hospitalized or on medical leave, although the difference did not reach significance (*p =* 0.058). In the multivariate analysis, the history of hospitalization or medical leave within the previous year was significantly associated with attrition, suggesting that the population who completed the study was in better shape, despite a nonsignificant difference in the diabetes-related micro- and macrovascular complication rates between groups (*p =* 0.587). In the German disease management program for type 2 diabetes, attrition was also associated with the presence of two or more secondary diseases (hypertension; stroke; lipid disorder; coronary heart disease; nephropathy; retinopathy; neuropathy; peripheral artery disease; blindness; myocardial infarction; amputation; diabetic foot; dialysis) but not with age (*p =* 0.348) [46]. Additionally, in the San Diego County Diabetes Program, worse clinical baseline conditions (higher blood pressure, HbA1c, and smoking habit) were found tobe associated with attrition but not sex or age [47]. On the other hand, younger age was also found to beassociated with attrition from diabetic care in Japan [48], and a Canadian study found that the major reason for attrition was the incompatibility between work schedule and center’s opening hours [49], which could be an explanation for the role of age, with older patients being retired.

On Reunion Island, there is a high proportion of low-income households [4], which was found to be associated with a higher attrition rate in this study. We did not find any association between the Universal Health Coverage "CMU" and attrition in the study, but CMU is an indirect indicator of socioeconomic status less indicative than household income. In the San Diego County Diabetes Program, the presence of insurance was a determinant of the attrition from the program [47]. The German program attrition was stratified on assurance status and did not conclude the effect of this factor [46]. We also found that patients living with another person were at higher risk of attrition than those living alone, although thoseliving with two were not. As people living with two persons were younger, it could be due to an interaction effect between age and the number of persons living home. These two findings are consistent with data from retrospective studies on defaulters from diabetes clinics [50].

The psychometric scales at baseline were not associated with attrition, although they included self-efficacy and outcome expectancy scales, which are the self-care activities the most consistently associated with regimen adherence, self-care behaviours and glycaemic control in T2DM patients [32, 51]. Finally, our results showed that 50% of the studied population presented underlying depression according to the CES-D scale and that this depressive status was significantly associated with attrition. The management of those psychological issues should be considered before or during intervention, as it increased the risk of attrition by a factor of 4. Depression is known to be significantly associated with nonadherence to diabetes treatment [52], increasing the risk of worse diabetes clinical outcomes among depressive patients.

The ERMIES nested qualitative study was published elsewhere [53]. The results are consistent with our quantitative analysis. The interviews performed in 44 patients at the beginning and 42 at the end of the trial analyzed self-care and disease management practices and their relationship with health literacy. It found that social support and the patient-provider relationship were important elements associated with a more interactive disease management posture. Interestingly, the five of 44 interviewed patients who belonged to the attrition group had great difficulty understanding and appraising health information, lower social support, and exhibited poor interactions with health care providers. This highlights the role of health literacy in achieving health practices, including medication adherence and disease monitoring [18, 54]. Health literacy is the result of a balance between individual skills and relationships with professionals, services and the health system. Low personal and social resources, burdensome family and social situations may hinder engagement with self-management [55].

The principal strength of the present study was the large baseline dataset with 140 variables (including sociodemographic, clinical, psychological, health care access, and lifestyle information) allowing us to draw a detailed image of the population included in the study and enabling an extensive analysis of potential factors associated with attrition.

Our work also had several limitations. First, this study was conducted in a small number of patients (*n* = 100) due to difficulties in patient enrolment and RCT interruption as the calculated necessary number of subjects (*n* = 240) was not obtained within a reasonable period (5 years). This denied the possibility of demonstrating any difference between the two groups in regard of HbA1c decrease (main outcome = 1% decrease in HbA1c at two years) due to lack of power and explains the decision of not publishing the RCT results. We thus have performed the analyses and prepared the study report. Second, our analyses were performed after multiple imputation, as only 58% of baseline data were complete. However, the proportion of missing data was low (< 5%) in the majority of variables, as shown in Table 1. The causes of missing observations or measurements were probably related to the important amount of paper questionnaires used in the trial.

## Conclusion

Our study presents several insights into baseline factors related to attrition in a trial testing the efficacy of a sustained self-management education intervention maintained over two years. The results are in favour of a higher risk of attrition in the most vulnerable (low income, recently hospitalized and depressive patients in particular) and in males. Considering these potential determinants and biases is important in scaling up interventions aimed at the optimization of long-term care in type 2 diabetes mellitus. Patient social vulnerability should be acknowledged in trials to focus specific actions to increase the retention rate and assess intervention efficacy.

## Data Availability

The datasets used and/or analysed during the current study are available from the corresponding author on reasonable request. Trial registration: ID_RCB number: 2011-A00046-35, Clinicaltrials.gov number: NCT01425866, (Registration date: 30/08/2011).

## Abbreviations

95% CI: 95% Confidence interval
BMI: Body mass index
CET: Compliance Evaluation Test
CMU: Universal Health Coverage
DQOL: BCI Diabetes Quality of Life, Brief Clinical Inventory
DSME: Diabetes self-management education and support
EGB: Permanent beneficiaries sample of French national health insurance database (Echantillon généraliste de bénéficiaires)
GP: General Practitioner
HADS: Hospital Anxiety and Depression Scale
HbA1c: Hemoglobin A1c
HLQ: Health Literacy Questionnaire
HR: Hazard Ratio
IQR: Interquartile range
MCA: Multiple correspondence analysis
MDQ: Multidimensional Diabetes Questionnaire
NEMT: Non-emergency medical transport
OAD: Oral antidiabetic drugs
RCT: Randomized controlled trials
T2DM: Type 2 diabetes mellitus
